# RELATIONSHIP SATISFACTION AND COGNITIVE WELL-BEING: FINDINGS FROM A DAILY DIARY STUDY

**DOI:** 10.1093/geroni/igae098.2878

**Published:** 2024-12-31

**Authors:** Selin Karakose, Martina Luchetti, Thomas Ledermann, Yannick Stephan, Antonio Terracciano, Angelina R Sutin

**Affiliations:** Florida State University, Tallahassee, Florida, United States; Florida State University, Tallahassee, Florida, United States; Florida State University, College of Education, Tallahassee, Florida, United States; University of Montpellier, Montpellier, Languedoc-Roussillon, France; Florida State University, Tallahassee, Florida, United States; Florida State University College of Medicine, Tallahassee, Florida, United States

## Abstract

Romantic relationships play a crucial role in many aspects of an individual’s life. Longitudinal research further supports the benefits of a satisfying relationship for multiple aspects of well-being. However, less work has examined these associations in daily life, particularly the association between relationship satisfaction and cognitive well-being. An analysis of relationship satisfaction in daily life is important to understand whether fluctuations in it may be associated with fluctuations in these aspects of well-being. This study examined the daily associations between relationship satisfaction and physical, psychological, and cognitive well-being. Every night for eight days, middle-aged participants in a committed relationship (N=303; 54% female; Mage=51.71, SD=7.32) reported on their relationship satisfaction and multiple aspects of well-being, including subjective cognition, as well as overall health, subjective age, purpose in life, and life satisfaction. Multilevel linear models examined within-person associations accounting for personal (sex, age, race, education), relational (relationship satisfaction), and contextual factors (day in the study, weekend day). On days when participants felt more satisfied in their relationships than their average, they also reported a sharper mind, better memory, and clearer thinking; relationship satisfaction was unrelated to whether participants were bothered and disrupted by forgetting. Relationship satisfaction was further related to feeling healthier, younger, more purposeful, and more satisfied with their life. These findings can inform relationship-based interventions to support better physical, psychological, and cognitive aging.

